# Production and Deformation of *Clonorchis sinensis* Eggs during In Vitro Maintenance

**DOI:** 10.1371/journal.pone.0052676

**Published:** 2012-12-20

**Authors:** Md. Hafiz Uddin, Young Mee Bae, Min-Ho Choi, Sung-Tae Hong

**Affiliations:** Department of Parasitology and Tropical Medicine, Seoul National University College of Medicine, Seoul, Republic of Korea; Queensland Institute of Medical Research, Australia

## Abstract

*Clonorchis sinensis* is a carcinogenic human liver fluke. The present study monitored eggs produced by long-term maintained adult worms of *C. sinensis* to confirm their egg productivity in vitro. The worms from infected rabbits were incubated in vitro in 1× Locke’s solution and broth media (RPMI-1640, DMEM and IMDM). Numbers of expelled eggs were counted sequentially and their morphological changes were monitored by microscopy after 1, 30, 60, and 90 days of cultivation. On the 1–3 days of cultivation, the eggs counted maximum 4,756±202 eggs/worm/day in IMDM medium. The number of eggs gradually decreased less than 1,000 at 7–14 days and below 100 at 21days but continued to pass eggs after 56 days in all media. Length of the eggs were reduced about 1 µm at 30 days, and the length/width ratio was maintained around 1.8 at 30 days but decreased to 1.7 at 60 days and 1.5 at 90 days. Faust-Meleney index (FMI) decreased as the cultivation duration increased and lowest FMI (5662.9±974.7) observed in IMDM media at day 90 (*P* = 0.001). Microscopic findings of the eggs recognized the miracidium in most of eggs at 60 days but not in those at 90 days. Instead, the eggs contained dark granules or vacuoles in the deformed shell at 90 days. Scanning electron microscopy revealed partial loss of wrinkles on the deformed egg surface and prominent abopercular knob. Eggs viability decreased as the cultivation progressed and showed significant positive correlation with FMI and length/width ratio. In conclusion, the cultivated worms pass only the eggs which are preformed in their uterus before cultivation. One gravid *C. sinensis* contains about 37,000 eggs in its uterus and produces about 4,000 eggs every day. The deformed eggs with FMI less than 7,000 and length/width ratio lower than 1.7 are non-viable.

## Introduction

Helminthes usually produce a large number of eggs to overcome the transmission barriers in nature. Production of enough number of eggs is essential for most flukes because they survive over 3 domains of hosts. The 3 host domains include the first snail intermediate host, the second intermediate host, and the definite host. Biological transition between the hosts is a physical or biological barrier for a fluke. When an adult fluke produces many eggs, only a few of the eggs may survive by successful invasion into the snail host, the first intermediate host. The infected snails shed cercariae into water after asexual reproduction cycles in their body, and the cercariae invade the second intermediate or definitive host to continue its life. Egg production is a fundamental biological function for reproduction of flukes.

*Clonorchis sinensis* is a liver fluke of human causing clonorchiasis, which is prevalent in East Asia. Clonorchiasis is now classified as one of food-borne nelgected tropical diseases. Since cholangiocarcinoma is a serious complication, clonorchiasis is a major health concern in endemic areas [1]. As egg production is an important process for a fluke’s biology and detection of eggs is a base of diagnosis, several studies observed egg productivity of *C. sinensis*. It has been reported that *C. sinensis* produce about 4,000 eggs/worm/day in humans, 2,400 eggs/worm/day in cats, and 1,600 eggs/worm/day in guinea pigs [2]. However, it is still unknown that *C. sinensis* may produce their eggs by in vitro cultivation.

Recently adult worms of *C. sinensis* were in vitro maintained long in broth media [3]. During the cultivation, the worms passed many eggs. The present study counted the eggs and observed morphological changes of eggs sequentially to investigate whether the worms are able to produce new eggs in broth media and whether they are viable.

## Materials and Methods

### Ethics Statement

The animal experiment was reviewed and approved by the institutional animal care and use committee of Seoul National University (2010).

### Collection of Adult Worms of *C. sinensis*

Metacercariae were collected from naturally infected fish *Pseudorasbora parva* according to the method described by Li et al. [4]. The collected metacercariae were preserved in cold (4°C) 1× PBS with antibiotics until use. The metacercariae were introduced to male New Zealand white rabbits and adult *C. sinensis* worms were recovered as described in our previous study [3].

### In vitro Cultivation of Adult Worms of *C. sinensis*

The fresh worms were distributed in a 6-well culture plate by 10 worms per culture well with 3 mL of test solution or media as described in the previous study [3]. All of the experiment procedures were performed in triplicates. The media included inorganic 1× Locke’s solution and 3 broth media: Roswell Park Memorial Institute-1640 medium (RPMI-1640), Dulbecco’s modified Eagle’s medium (DMEM), and Iscove’s modified Dulbecco’s medium (IMDM) with penicillin 100 µg/mL and streptomycin 100 U/mL concentrations. The 1× Locke’s solution contained NaCl 8.9 g, KCl 0.42 g, NaHCO_3_ 0.2 g, and CaCl_2_ 0.24 g (wt/vol) in 1 liter of distilled water. The broth media were purchased from the WelGENE (Seoul, Korea). The culture solution or media were refreshed in every 3 days. The worms were cultivated in a humidified incubator at 37°C in the presence of 5% CO_2_. Some of this experiment procedure was shared with the in vitro cultivation of *C. sinensis* adults [3].

### Egg Counts Expelled by Adult Worms

The wells of the 6-welled culture plates were cleaned properly before the collection of eggs. The eggs expelled by the adult worms for 24 hours in different media were collected from the wells by washing 3 times with respective medium. Numbers of eggs were counted after 1, 3, 7, 14, 21, 28, 35, 42 and 56 days, and the numbers of eggs/worm/day were estimated.

### Measurement of Eggs under Light Microscopy

Eggs collected at 1, 30, 60, and 90 days of incubation were randomly measured for their length and width (n = 15) with a microscope (Olympus, Japan) at ×400 magnification, 0.29 resolving power with a resolution of 1,360×1,024 pixels (digital image) using Image-Pro® Express software version 4.0.1. For better understanding the dimensions of eggs and comparison, eggs obtained from different solution and media at different duration were evaluated by the length and width ratio (L:W) as well as Faust-Meleney Index (FMI = L×W^2^; where L, length and W, width) [5]. The eggs were also observed for internal contents such as miracidium, vacuolated globules, dark mass as well as prominence of abopercular knob using light microscope with ×100 or ×400 or ×1000 magnifications.

### Scanning Electron Microscopy

For scanning electron microscopy (SEM) of eggs, long-term cultivated worms from 1× Locke’s solution and different broth media were fixed in 10% neutral buffered formalin and in 1% osmium tetroxide (OsO_4_) at 4°C. After dehydration through graded series of ethanol (50%–100%), the specimens were dried using a critical-point dryer. The dried adult worms were mounted on aluminum stubs and their body was dissected to open upper uterine branches which were full of eggs. The uterine eggs were coated with gold-plated metal supports. Ultrastructures of the egg shell surface and abopercular knob were observed by scanning electron microscope ABT DS-130C, Japan with ×2000 or ×4000 magnification. Normal *C. sinensis* eggs from freshly collected worms were also evaluated by SEM for the comparison of abopercular knob.

### Determination of Egg Viability

Viability of *C. sinensis* eggs was determined by trypan blue (0.4%) staining. Eggs from different solution and media were mixed with equal volume of trypan blue and kept in room temperature for about 10 minutes. The eggs then examined under light microscope and the unstained one was counted as viable. Prior to the experiment eggs were stained with different kinds of dye namely eosin Y (0.1%), lugol’s iodine (1%), methylene blue (0.01%) and trypan blue (0.4%) to select the best (Supporting Information: Materials and Methods; Figure S1).

### Statistical Analysis

All the data analyzed using Microsoft office excel 2007 program. Student’s t-test with two tails was performed and the Pearson’s correlation coefficient was determined for comparison and difference at *P*<0.05 was regarded as significant.

## Results

### Egg Counts in Different Media

Egg counts varied depending on the media and duration of cultivation (Table 1). The worms in IMDM produced 4,756±202 eggs/worm/day on day 1, which was the highest among the solution and media. At day 3, the worms in RPMI-1640 produced significantly more eggs of 4,480±257 than those in other media (*P*<0.001). In the inorganic 1× Locke’s solution, a different behavior of egg expulsion was noticed, starting from low count of 220±44 at day 1 to a peak of 2,827±433 at day 7. The egg counts decreased to 1,317±113 after 14 days, and less than 200 after 28 days. Few worms had eggs in their uterus afterwards in all media.

**Table 1:** Egg counts of *C. sinensis* daily produced by one adult worm in different media during the cultivation.

Solution/Media	No. of eggs/worm/day (mean ± SD) at different day(s)									
	1	3	7	14	21	28	35	42	56	Appx. Total*
1× Locke's	220±44	1023±64	2827±433	1317±113	143±33	296±34	128±3	88±8	194±37	38940±3443
RPMI-1640	2887±219	4480±257	1142±152	317±50	217±57	16±5	99±10	22±4	21±4	36571±1026
DMEM	4273±152	1964±374	2529±228	437±64	234±41	43±7	40±8	22±4	11±2	39705±311
IMDM	4756±202	2853±240	1107±189	456±99	116±17	45±12	10±3	56±4	37±10	33881±2254

SD = standard deviation.

* Approximate total number of egg estimated by assuming same count of egg during intervals.

### Measurements of Eggs under Light Microscope

Table 2 summarized measurements of the discharged eggs in culture media. Since the worms survived up to 60 days in 1× Locke’s solution, data were available till day 60. The egg length decreased significantly at day 30 and 60 in 1× Locke’s solution and at day 30, 60, and 90 in RPMI-1640 and IMDM media (*P*<0.001). Average width of the egg showed variability among the solution and media. In 1× Locke’s solution it showed significant reduction from 16.22±0.65 at day 1 to 15.14±0.24 at day 30 (*P*<0.001), however, it was increased slightly in the broth media but not significant. The ratio of length and width of eggs decreased significantly during the cultivation among the broth media (*P*<0.001). The eggs in the Locke’s solution showed a significant decrease of the length/width ratio from 1.81±0.11 at day 1 to 1.74±0.05 at day 60 of incubation as shown in Table 2 (*P*<0.001). FMI value decreased significantly between day 1 (7699.3±707.4) and day 30 (6361.2±382.2) in case of 1× Locke’s solution and between day 1 (6908.0±301.6) and 90 (5662.9±974.7) in case of IMDM media (*P* = 0.001). RPMI-1640 and DMEM also showed a lower FMI value at day 90 compared to day 1, however, it was not significant (*P* = 0.064 and *P* = 0.203 for RPMI-1640 and DMEM respectively).

**Table 2:** Measurements of eggs obtained from different media after cultivation (n = 15).

Parameters	Media	Day(s) of cultivation			
		1	30	60	90
Length (µm)	1× Locke's	29.23±1.36	27.73±1.20	27.49±0.48	ND
	RPMI-1640	28.32±0.97	27.88±0.69	28.11±2.59	22.84±2.04
	DMEM	28.92±0.93	27.98±1.17	26.43±2.45	24.91±2.61
	IMDM	29.58±0.73	27.98±0.91	27.79±0.89	23.99±1.60
Width (µm)	1× Locke's	16.22±0.65	15.14±0.24	15.77±0.32	ND
	RPMI-1640	15.39±0.40	15.40±0.37	16.03±0.95	15.81±1.07
	DMEM	15.44±0.49	15.30±0.58	16.21±1.06	16.06±0.95
	IMDM	15.28±0.29	15.90±0.32	15.77±0.74	15.31±1.03
Length/Width ratio	1× Locke's	1.81±0.11	1.83±0.08	1.74±0.05	ND
	RPMI-1640	1.84±0.06	1.81±0.08	1.76±0.19	1.44±0.06
	DMEM	1.87±0.07	1.83±0.09	1.64±0.20	1.56±0.18
	IMDM	1.94±0.06	1.76±0.07	1.77±0.09	1.57±0.12
FMI	1× Locke's	7699.3±707.4	6361.2±382.2	6839.4±299.6	ND
	RPMI-1640	6719.0±511.4	7251.8±1124.5	6608.6±267.6	5794.0±1393.9
	DMEM	6904.3±542.9	6957.8±1006.1	6557.9±594.9	6443.5±961.3
	IMDM	6908.0±301.6	6926.4±721.1	7073.1±342.1	5662.9±974.7

ND = No data, FMI: Faust-Meleney index.

### Microscopic Changes of the Eggs

**Figure 1:**
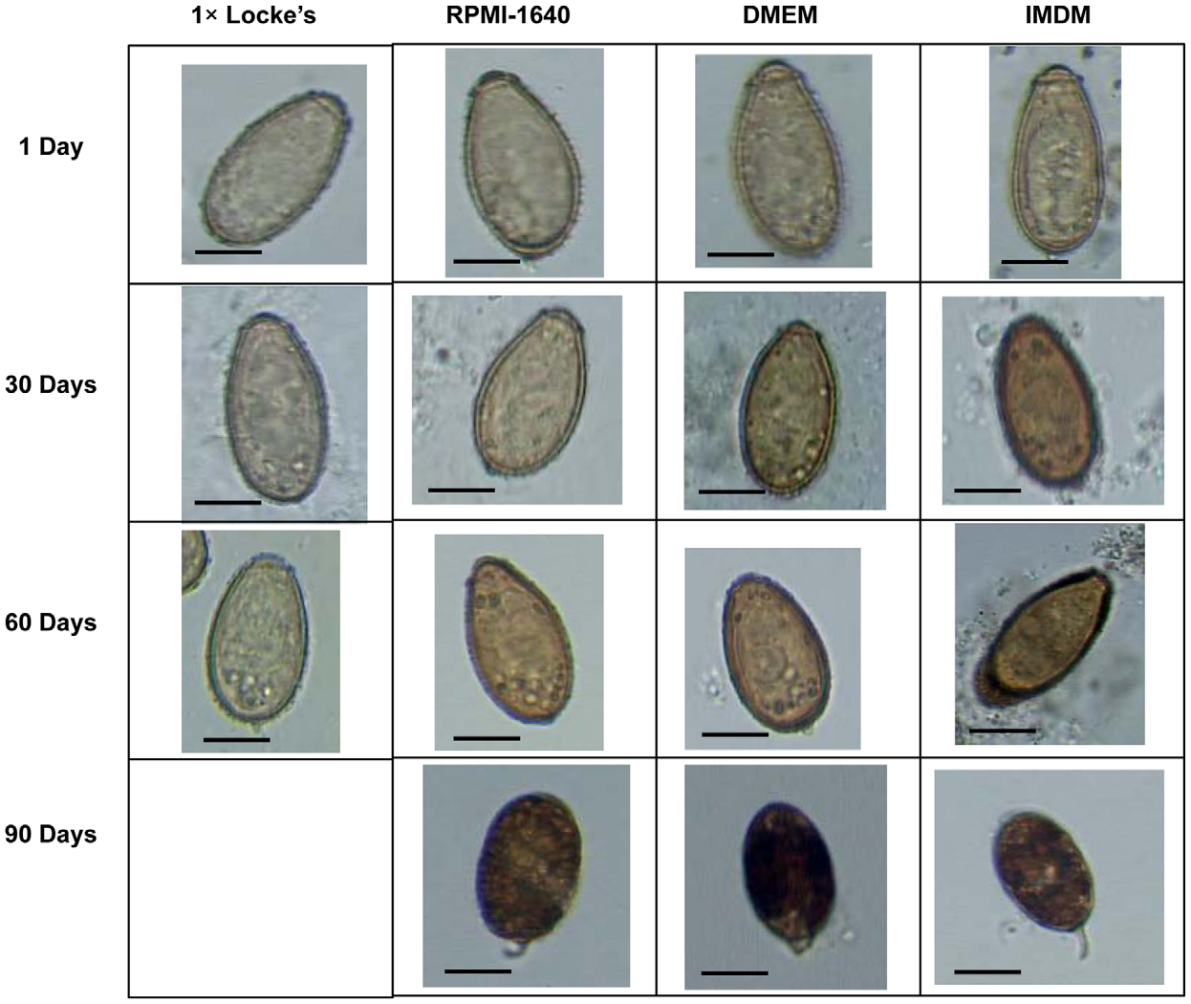
Light microscopic changes of *C. sinensis* eggs in different solutions and media during cultivation. Morphological view of eggs from day 1 to day 90 arranged from top to bottom. As the worm survived up to 60 days in 1× Locke’s solution there was no data for day 90. All the eggs showed typical morphology at day 1, however, at day 90 eggs showed atypical shape, shorter length, prominent abopercular knob along with interior dark granules (×400). Scale bar: 10 µm.

**Figure 2:**
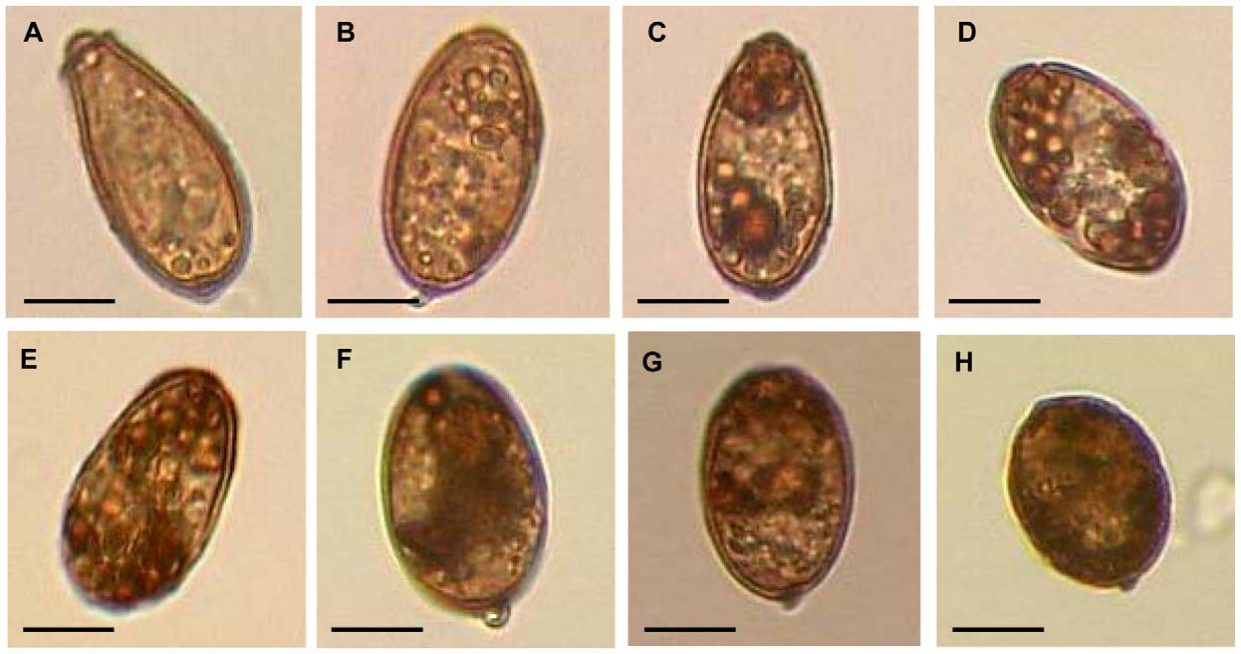
Deformed *C. sinensis* eggs observed after 90 days of incubation in different broth media. (A) Normal egg. (B) Egg containing deformed granules. (C) Egg containing dark granules both at opercular and abopercular ends. (D) Dark granules fragmented in to smaller granules. (E) Fragmented dark granules covered whole interior part of the egg. (F) Degraded dark granules in side the egg with prominent abopercular knob. (G) Egg with deformed shape and degraded miracidium. (H) Extremely deformed egg with degraded larval products. Original magnification ×400. Scale bar: 10 µm.

**Figure 3:**
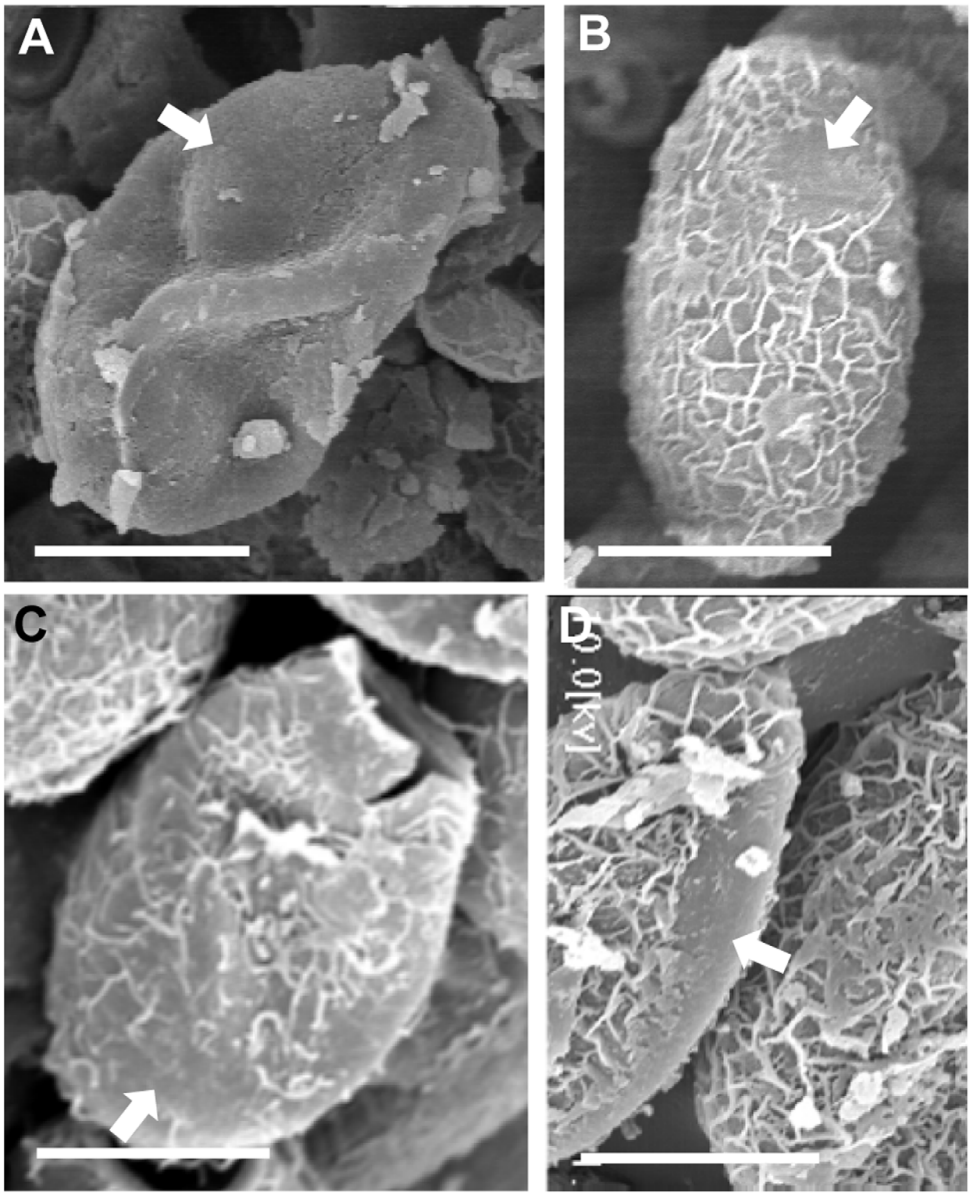
Surface ultrastructure of *C. sinensis* eggs by scanning electron microscopy. Eggs after 60 days of incubation in 1× Locke’s solution (A) and 90 days of incubation in RPMI-1640 (B), DMEM (C) and IMDM (D) culture media (×2000). Arrow head showed smoothness on the surface or loss of wrinkles. Scale bar: 10 µm.

**Figure 4:**
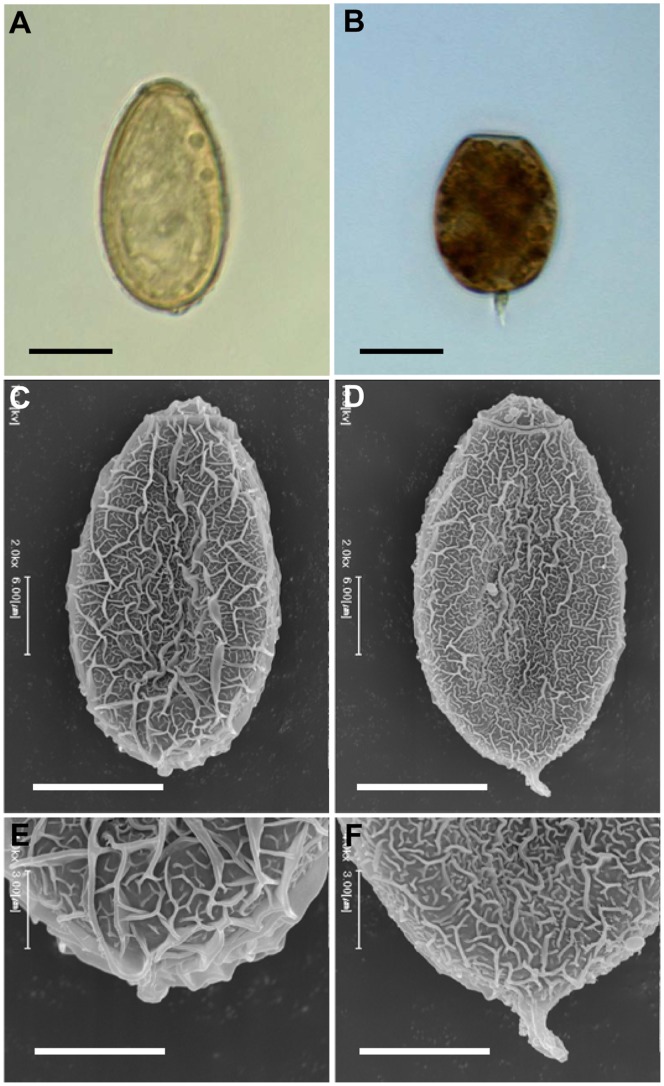
Abopercular knob of normal and cultured *C. sinensis* eggs. (A) Light microscopy showed an inconspicuous abopercular knob in a normal egg (×1000). (B) Very prominent abopercular knob in cultured worm’s egg (×1000). (C–D) SEM images of normal and cultured worm’s eggs respectively (×2000). (E) Magnified posterior portion of normal egg (×4000). (F) Posterior portion of cultured worm’s egg with distinct abopercular knob (×4000). Scale bar: 10 µm for A–D and 5 µm for E–F.

Light microscopy observed gradual morphological changes in the eggs from day 1 to 90 (Figure 1). All of the eggs were clean and distinctive of normal configuration on day 1. A few eggs included vacuoles on day 30 and both of number and size of the vacuoles increased on day 60. Most of the eggs discharged in the media or in the uterus of the worms were full of vacuoles or dark granules in their deformed egg shell observed at day 90 as presented in Figure 2. SEM observed surface topography of the eggs collected from 1× Locke’s solution at day 60 and from other broth media at day 90. Partial loss of surface wrinkles was evident among the eggs observed in 1× Locke’s solution and different media (Figure 3). Most of the eggs at day 90 demonstrated long prominent abopercular knob (Figure 4).

### Viability of the Eggs

Almost all the eggs were viable among the solution and media collected on day 1, however, viability decreased gradually according to the duration of cultivation (Table 3). On day 30 and 90 eggs from RPMI-1640 showed highest viability (79.1% ±1.11% and 34.3% ±0.4% respectively) but on day 60, highest viability (67.8% ±0.7%) was observed in 1× Locke’s solution. The egg viability showed a significant positive correlation with FMI values (1× Locke’s: *r* = 0.987, n = 6, *P*<0.001; RPMI-1640: *r* = 0.671, n = 8, *P* = 0.034; DMEM: *r* = 0.697, n = 8, *P* = 0.027 and IMEM: *r* = 0.803, n = 8, *P* = 0.008). Correlation was also significant for length and width ratio except for 1× Locke’s solution (RPMI-1640: *r* = 0.736, n = 8, *P* = 0.018; DMEM: *r* = 0.805, n = 8, *P* = 0.007 and IMEM: *r* = 0.949, n = 8, *P*<0.001).

**Table 3:** Proportion of viable eggs in different solution and media during cultivation determined by trypan blue (0.4%).

Solution/Media	Proportion of viable eggs (%) by day(s)			
	1	30	60	90
1× Locke's	99.1±0.1	75.5±1.9	67.8±0.7	ND
RPMI-1640	98.8±0.2	79.1±1.1	38.3±1.0	34.28±0.4
DMEM	99.6±0.1	52.0±2.8	44.3±3.7	25.39±2.5
IMDM	99.5±0.2	69.2±1.9	43.2±0.4	17.85±0.3

ND = No data.

## Discussion

Adult *C. sinensis* worms which were incubated in broth media produced more than 4,000 eggs/worm/day at the beginning. Thereafter, the egg counts decreased to just above 1,000 at day 7, and to 300–400 at 14 day. The counts became lower than 100 at 28 day in the broth media. The counts were similar to each other among 3 broth media. Contrary to this, the counts in the Locke’s soultion were 220±44 and 1023±64 at day 1 and 3 respectively. These counts were lower than those in broth media at the beginning, but the worms produced more eggs at day 21 and later. The worms in broth media produced their eggs more vigorously at early phase of incubation while those in the Locke’s solution produced less eggs daily but for a longer period. The daily egg counts of 4,000 by one adult worm were compatible with the estimated mean number of 3,770 eggs from an infected human [2].

Based upon the egg counts, total eggs produced by an incubated adult worm, which was recovered from rabbits after 8 weeks of infection, were estimated 37,274±2,988 in average. These total egg counts were similar regardless of the cultivation solution or media. The daily egg counts decreased rapidly and the uterus became empty during the cultivation. Therefore, the present daily egg counts represented that the in vitro cultivated worms produced only the intrauterine eggs which were preformed in their uterus while they were living in rabbits before the cultivation. Based on the data, it is estimated that one gravid *C. sinensis* contains about 37,000 eggs in its uterus and produces about 4,000 eggs every day. Accordingly, the eggs may stay in the uterus around 10 days for intrauterine maturation. Most of the eggs in the uterus were found mature and the miracidium developed already, but some of them were immature mainly in the proximal uterus. Those immature eggs failed to develop further during the cultivation. These findings suggest that the cultivated worms are unable to formulate new viable eggs and maturation of preformed immature eggs is also impossible by in vitro cultivation.

*C. sinensis* eggs range 28–31×13–16 µm in size, having an operculum at its conical end and a small abopercular knob [6]. In the present study, the expelled eggs showed abnormal contour as the worm survived longer. The average length of the egg was reduced gradually and significantly in the Locke’s solution (27.49±0.48 µm) and all broth media. The length reduction resulted in reduction of the FMI value. The FMI was proposed as a parameter for biometric analysis to approximate volume of eggs for heterophyid and opisthorchiid flukes [5]. In the present investigation, the FMI values decreased at day 60 in the Locke’s solution and at day 90 in broth media. The decreased FMI values suggest reduction of egg volume by shrinkage of length and degeneration of the contents. Most of the eggs produced after 60 days of in vitro cultivation were deformed, and their length/width ratio was less than 1.7 and the FMI value was less than 7,000. Most of those eggs were determined non-viable when stained with trypan blue. Cultivation for 60 days might be the maximum period of egg viability.

Light microscopic observation revealed accumulation of vacuoles and dark masses inside the egg shell as the cultivation lasted longer than 60 days. The vacuoles and dark masses seemed to be deformed vitelline cells as shown in *O. viverrini* [7]. Those materials in the eggs suggested that vitelline cells or other egg forming materials were degenerated and the eggs were abortive. The mature eggs which form the miracidium may survive long but aborted ones do not. The degeneration of inner materials might have induced deformity and reduction of egg volume. A study found deformed eggs when mature *Echinostoma caproni* gravid adults stored in Locke’s solution up to 4 months at 4°C [8]. One possible explanation for above changes may be improper quinone tanning which is associated to the process of egg shell formation [9]. Long- term in vitro cultivation may have affected this phenol-oxydase mediated enzymatic process resulting deformed egg shell [10]. Thus prolonged in vitro cultivation of *C. sinensis* may deform the eggs as well as their viability.

Mature eggs of *C. sinensis* contain a very inconspicuous abopercular knob. *O. viverrini,* a closely related liver fluke, also shows very small to slightly developed abopercular knob of the eggs [7]. In the present study, abopercular knob of the egg shell became more prominent in most of the eggs after 60 days of incubation. The appearance of such a prominent abopercular knob was obvious in most degenerated eggs. Krejci and Fried [11] demonstrated that abopercular knob can be shallow or deeply infolded in the egg shell in case of *Echinostoma caproni* and *E. trivolvis* respectively. Infolding of abopercular knob surrounding shell was not observed in the present study. The prominent abopercular knob may be an extension of existed normal inconspicuous knob in response to the changing physiology of dying worms. Taken together, it is difficult for the flukes to produce normal viable eggs by in vitro cultivation.

The surface structure of eggs, observed by SEM, appeared to be a suitable morphological feature for understanding status of the egg shell. Among opisthorchiids, *C. sinensis* egg surface is extremely sculptured to make interlaced wrinkles of varying height which helps the ability to accumulate natural aquatic fibers and to increase the possibility of contact with molluscan hosts [12], [13]. In the present study, diminished surface wrinkle was observed by SEM of the aborted eggs. This diminished wrinkling with the prominent abopercular knob may be an outcome of degenerating process of the egg shell.

The present study had a few limitations. There were no direct data of egg viability to compare with morphological changes. In vitro hatching of eggs can give accurate information to the egg viability, but that of *C. sinensis* eggs is still unable. Also data of morphology and physiology of the incubated worms were limited.

In conclusion, the cultivated *C. sinensis* worms in broth media pass viable eggs for 60 days which are preformed in their uterus during their survival in rabbits. The preformed immature eggs are unable to develop miracidium by in vitro cultivation. One mature *C. sinensis* adult contains about 37,000 eggs in the uterus and produces about 4,000 eggs daily. The deformed eggs of the FMI value less than 7,000 and length/width ratio lower than 1.7 are non-viable.

## Supporting Information

File S1 **Determination of** ***C. sinensis*** **egg viability using different kind of dyes.**
(DOC)

Figure S1 **Staining of** ***C. sinensis*** **egg for the determination of viability with different types of dye.** The upper and lower rows showed viable and non-viable eggs respectively. Trypan blue (0.4%) and eosin Y (0.1%) selectively stained the non-viable eggs where as lugol’s iodine (1%) and methylene blue (0.01%) stained both viable and non-viable eggs. Scale bar: 10 µm.
(TIF)
