# Supplementary material for: Production and Deformation of Clonorchis sinensis Eggs during In Vitro Maintenance
Source: PLoS One. 2012 Dec 20;7(12):e52676. doi: 10.1371/journal.pone.0052676 (PMC3527588; doi:10.1371/journal.pone.0052676)
Supplement: File S1 — Determination of C. sinensis egg viability using different kind of dyes. (DOC) [file pone.0052676.s001.doc]

**Determination of *C. sinensis* egg viability using different kind of dyes.** Egg viability was checked with different dyes namely eosin Y (0.1%), lugol’s iodine (1%), methylene blue (0.01%) and trypan blue (0.4%) (Figure S1). The eggs of *C. sinensis* which were collected on day 1 and preserved in 4°C considered as positive control or viable eggs. For the preparation of non-viable eggs, eggs were kept in -20°C for 24 hours then thawed in room temperature followed by heating in 70°C for 10 minutes in a water bath. The eggs were mixed with dyes at 1:1 ratio and kept in room temperature for 10 minutes. Unstained and stained eggs were regarded as viable and non-viable respectively. Lugol’s iodine and methylene blue were found to stain both viable and non-viable eggs. Trypan blue showed the best differentiation among the dyes and was used for the determination of egg viability.
