# Supplementary figures and images for: Production and Deformation of Clonorchis sinensis Eggs during In Vitro Maintenance
Source: PLoS One. 2012 Dec 20;7(12):e52676. doi: 10.1371/journal.pone.0052676 (PMC3527588; doi:10.1371/journal.pone.0052676)

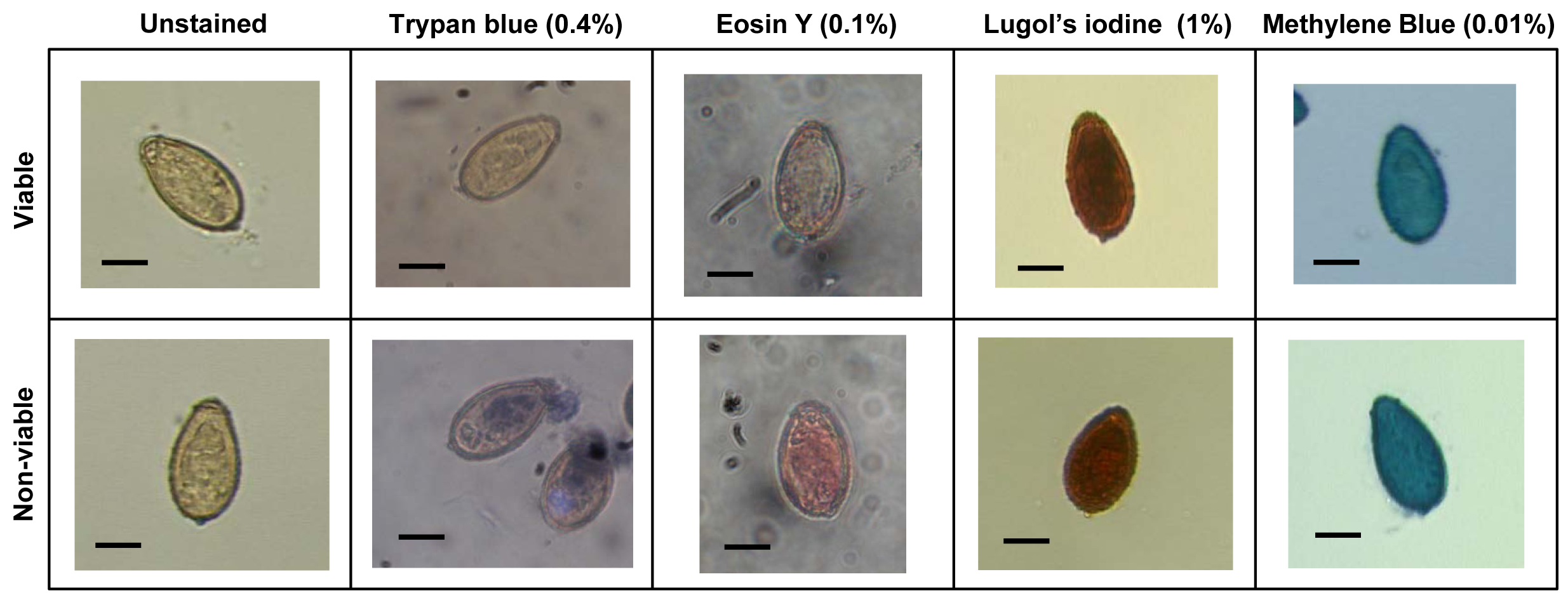

Supplement: Figure S1 — Staining of C. sinensis egg for the determination of viability with different types of dye. The upper and lower rows showed viable and non-viable eggs respectively. Trypan blue (0.4%) and eosin Y (0.1%) selectively stained the non-viable eggs where as lugol’s iodine (1%) and methylene blue (0.01%) stained both viable and non-viable eggs. Scale bar: 10 µm. (TIF) [file pone.0052676.s002.tif]
